# Flexible and Stretchable Carbon-Based Sensors and Actuators for Soft Robots

**DOI:** 10.3390/nano13020316

**Published:** 2023-01-12

**Authors:** Xinyi Zhou, Wenhan Cao

**Affiliations:** 1 School of Information Science and Technology, ShanghaiTech University, Shanghai 201210, China; 2 Shanghai Engineering Research Center of Energy Efficient and Custom AI IC, Shanghai 201210, China

**Keywords:** flexible electronics, stretchable electronics, carbon nanotubes, graphene, sensors, actuators, soft robotics

## Abstract

In recent years, the emergence of low-dimensional carbon-based materials, such as carbon dots, carbon nanotubes, and graphene, together with the advances in materials science, have greatly enriched the variety of flexible and stretchable electronic devices. Compared with conventional rigid devices, these soft robotic sensors and actuators exhibit remarkable advantages in terms of their biocompatibility, portability, power efficiency, and wearability, thus creating myriad possibilities of novel wearable and implantable tactile sensors, as well as micro-/nano-soft actuation systems. Interestingly, not only are carbon-based materials ideal constituents for photodetectors, gas, thermal, triboelectric sensors due to their geometry and extraordinary sensitivity to various external stimuli, but they also provide significantly more precise manipulation of the actuators than conventional centimeter-scale pneumatic and hydraulic robotic actuators, at a molecular level. In this review, we summarize recent progress on state-of-the-art flexible and stretchable carbon-based sensors and actuators that have creatively added to the development of biomedicine, nanoscience, materials science, as well as soft robotics. In the end, we propose the future potential of carbon-based materials for biomedical and soft robotic applications.

## 1. Introduction

Due to the superior softness and elasticity compared to conventional rigid silicon-based semiconductor devices, flexible and stretchable electronics have received great attention in the past few decades [1,2], especially in the community of electrical and computer engineering [3,4], materials science [5,6], chemistry [7,8], biology and biomedical engineering [9,10,11]. Devices manufactured from flexible materials or structures have the mechanical capability to intrinsically bend, twist, stretch, and compress while maintaining excellent electrical properties and working performance, which has greatly expanded the applications and opened up new opportunities for various novel electronic devices [12,13]. In particular, the rapid development of synthetic chemistry and materials science has led to a huge number of polymeric materials being devised and employed as substrates for fabricating flexible devices, such as polydimethylsiloxane (PDMS) [14,15], polyimide (PI) [16,17], polyethylene terephthalate (PET) [18,19], and hydrogel-based materials [20,21]. Additionally, the introduction of conducting polymer composites, such as poly(3,4-ethylenedioxythiophene):poly(styrenesulfonate) (PEDOT:PSS), as well as other organic semiconducting materials, has impressively made possible intrinsically stretchable transistors and thus logic circuits [22,23,24,25]. In addition to the aspect of material selection, the structural optimization of devices, such as wavy [26,27], kirigami and origami structures [28,29], and mesh structure configuration [30,31], also greatly contribute to bulk material stretchability and sensitivity for flexible sensors by adding to the degrees of freedom of individual sensing units [32,33]. Since such electronic devices retain outstanding properties of flexibility, biocompatibility, stability, and robustness [34,35], they have extended to several application fields including wearable devices [36,37], soft robotics [38,39], and energy harvesting [40,41], where such devices make up for the defects of stiff electronic devices.

Carbon-based materials have gradually grown prominent in the field of study for their excellent biocompatibility, wearability, and conductivity while being integrated together with polymers or elastomers as composite substrates [42,43,44]. It has been shown at the beginning of this century that carbon nanotubes can be constituents for flexible transistors and electrodes owing to their outstanding properties in carrier mobility, electrical conductivity, surface area, and versatility [45,46]. Researchers have now expanded the scope of their study to various low-dimensional carbon nanomaterials and their derivatives, such as graphene, reduced-graphene oxide, and carbonized films [47,48]. Different types of carbon nanomaterials possess distinctively different mechanical and physical properties and are applied in diverse fields. Two of the most significant applications are sensors and actuators, which interestingly implement the conversion of electric signals and other forms of energy in opposite ways between the environment and the input/output units of the system. Flexible carbon-based electronic devices are considered to take advantage of both carbon materials and soft substrates so that they have innovative applications, such as flexible piezoresistive electronic skin [49], self-healing strain sensor for monitoring organism movement [50], and carbon-coated substrates for biomedical sensing and implant [51]. These applications pave the way for state-of-the-art flexible and stretchable wearable devices, as well as soft robotics.

In this review, we begin with an introduction of applying carbon-based materials to flexible and stretchable electronics in Section 1. Then in Section 2, the synthetic methods of three commonly used low-dimensional carbon materials, namely carbon dots, carbon nanotubes, and graphene, as well as the fabrication methods of merging them with polymers, are discussed in detail. We further summarize the recent progress of carbon-based flexible devices in terms of soft robotic sensors and actuators in Section 3 and Section 4, respectively. Representatives of sensing and actuating for different purposes are categorized and described so as to demonstrate the significance of employing carbon-based materials. Finally, in Section 5, we present the potential challenges of flexible and stretchable devices based on carbon nanomaterials and give an outlook of the potential future novel devices.

## 2. Materials Synthesis and Fabrication

During the discovery of materials for soft robotic devices, in addition to carbon nanomaterials, numerous low dimensional materials have been explored in depth, such as transition metal dichalcogenides (TMDCs) [52], nanowires (NWs) [53], metal films [54], and colloidal quantum dots (QDs) [55]. TMDCs, as layered two-dimensional materials, have fascinating electronic and optical properties, rendering TMDCs especially popular in sensing applications. In particular, MoS_2_, MoSe_2_, WS_2_, and WSe_2_ are common TMDCs and were discovered to be well-suited for transistors, sensors, inverters, and photodetectors [56,57,58]. NWs used to be assembled in functional electronic devices, and now their benefits to flexible electronics have been revealed [59]. One of the most regularly used types is silver nanowire (AgNW) due to its high conductivity and transmittance as well as the stretchability acquired from its geometry and low dimensionality, resulting in its wide use in flexible electrodes, wearable strain and temperature sensors [60,61,62]. Other than that, flexible transparent electrodes can be conveniently and inexpensively fabricated with deposited metal films and stretchable substrates, provided that metallic thin films perform better than indium tin oxide (ITO) in cost and stability [63,64]. As colloidal QDs excel in electronic and optical properties, they are advisable for electronic and optoelectronic devices with low cost, large area and good flexibility [65,66].

However, among materials used in flexible devices, carbon-based materials are reported to have advantages in affordability, simplicity in fabrication and extraordinary functional performance. Their superiority makes them widely used for realizing flexibility and good performance simultaneously, with relatively low cost, non-toxicity, promising mechanical and electronic properties [67,68]. Carbon-based materials are derived from such a huge family that numbers of materials have been detected and synthesized on the basis of the element carbon, including various forms of carbon nanotubes [69], carbon onions [70], carbon nanohorns [71], graphene [72], and carbon dots [73], particularly since the first discovery of buckminsterfullerene in 1985 [74].

In this section, we introduce three major basic low dimensional carbon-based materials, namely zero-dimensional (0D) material carbon dots, one-dimensional (1D) material carbon nanotubes, and two-dimensional (2D) material graphene, as well as discussing the synthesis of each nanomaterial and the fabrication methods of integrating them with soft substrates. Since these three carbon-based materials share some common synthetic approaches, Table 1 is presented to compare the differences of several synthetic methods, where their advantages and drawbacks are listed.

### 2.1. Carbon Dots

Carbon dot is a 0D nanostructure with characteristic size lower than 10 nm [88], accidentally discovered by Xu et al. during the procedure of purifying single-walled carbon nanotubes in 2004 [73]. In contrast with some typical III-V or II-VI semiconductor quantum dots involving heavy metals, such as CdSe, carbon dots are greener and safer when in contact with biomass [89]. Therefore, carbon dots have engaged in a wide range of biological and biochemical applications in view of their excellent properties in aspects of toxicity, chemical inertness, and biocompatibility [90,91].

**Figure 1:**
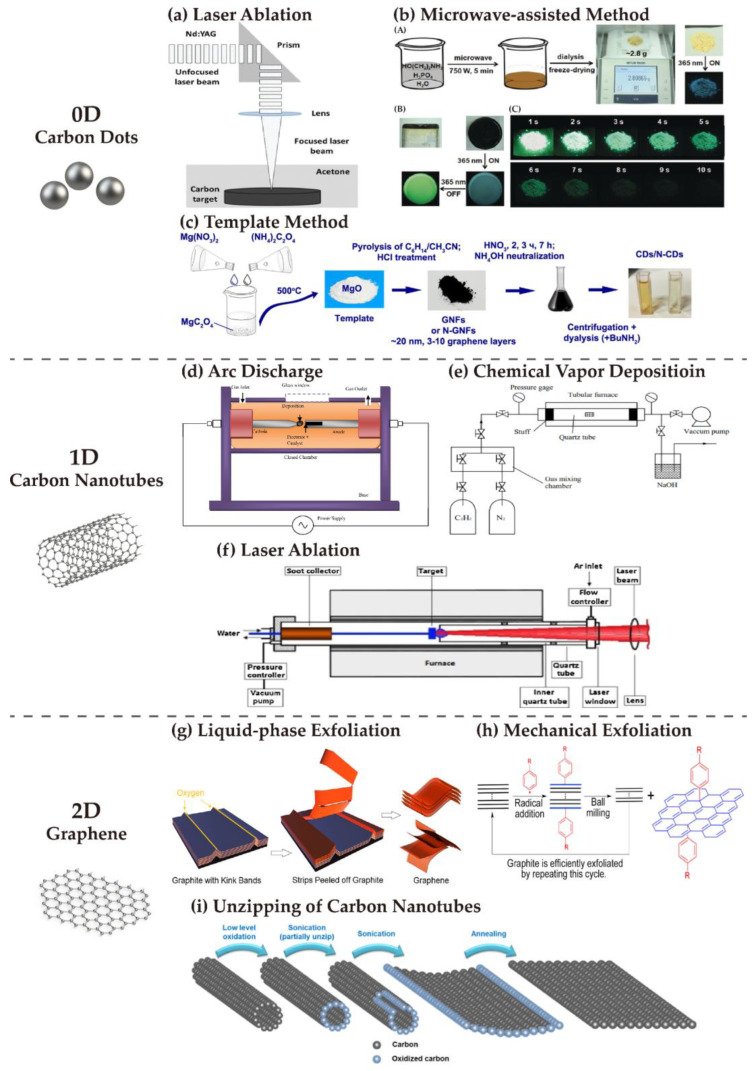
Schematic of typical fabrication techniques for 0D, 1D, and 2D carbon–based materials. (**top**) Carbon dots. (**a**) Figure for laser ablation: Reproduced from [75] with permission, copyright 2016, Springer Nature. (**b**) Figure for microwave–assisted method: (**A**) Schematic of the preparation process and purification of carbon dots; (**B**) Images of the crude product under different irradiation conditions; (**C**) Images of purified carbon dots after ceasing UV irradiation. Reproduced from [94] with permission, copyright 2018, John Wiley and Sons. (**c**) Figure for template method: Reproduced from [81] with permission, copyright 2019, Elsevier. (**middle**) Carbon nanotubes. (**d**) Figure for arc discharge: Reproduced from [78] with permission, copyright 2014, Elsevier. (**e**) Figure for chemical vapor deposition: Reproduced from [82] with permission, copyright 2018, Elsevier. (**f**) Figure for laser ablation: Reproduced from [76] with permission, copyright 2015, John Wiley and Sons. (**bottom**) Graphene. (**g**) Figure for liquid–phase exfoliation: Reproduced from [83] with permission, copyright 2020, ACS. (**h**) Figure for mechanical exfoliation: Reproduced from [84] with permission, copyright 2019, Elsevier. (**i**) Figure for unzipping of carbon nanotubes: Reproduced from [86] with permission, copyright 2021, ACS.

The fabrication techniques of carbon dots can be generally categorized into top-down and bottom-up approaches. In particular, top-down approaches reduce larger bulk materials to smaller sophisticated shapes and structures, including arc discharge, laser ablation, and electrochemical or chemical oxidation; in contrast, bottom-up approaches convert or assemble smaller building block structures into carbon dots, in which thermal, microwave-assisted, and template are instances of methods [77,92,93]. The schematic diagrams of some mentioned typical synthetic approaches are shown in Figure 1a–c.

Carbon nanotube-derived carbon dots were acquired through oxidizing arc-discharge soot early in 2004 [73], followed by laser ablation being proposed in 2006, where the carbon target was exposed in water vapor and the carrier gas was argon [95]. Subsequently, there appeared to be a breakthrough in bottom-up methods. Carbon dots were first obtained from combustion by Liu et al. in 2007, in which organic objects, such as ethanol and candle, were burned in the first place. Then the soot from burn was collected, and carbon dots could be generated though oxidation treatment [96,97]. Additionally, the diverse properties of carbon dots, in terms of emitting fluorescence and reaction to specific substances, can be decided by burning from different precursors [98,99,100].

### 2.2. Carbon Nanotubes

After the discovery of fullerene in 1985, this C_60_ species was expected to have high chemical and practical value [74]. In 1991, carbon nanotubes (CNTs), were discovered by Iijima, which are 1D carbon nanomaterials based on the fullerene structure [69,101]. CNTs can be made from rolling graphite sheets in a few different ways to realize the diverse atomic arrangement and size of the tube. Therefore, CNTs possess distinctive mechanical and electronic properties [102]. They can be classified into three types by the number of layers, which were named as single-walled carbon nanotubes (SWCNTs), double-walled carbon nanotubes (DWCNTs), and multi-walled carbon nanotubes (MWCNTs) [103]. Additionally, according to the rolling orientation of CNTs, SWCNTs can be further categorized by chirality, namely zig-zag, chiral, and armchair form [104]. Considering their outstanding behavior in tensile strength, electrical conductivity and thermal conductivity resulted from the 1D structure and covalent bonding, devices containing CNTs are supposed to have favorable stretchability, durability and reaction speed [105,106].

Figure 1d,f show that CNTs share a few similar synthetic methods with carbon dots, such as arc discharge and laser ablation. In the arc discharge evaporation method, two graphite electrodes, both connected to the power supply, are placed in a reactor with helium. When the reactor is heated to 4000 K and the power is switched on, carbon starts to vaporize, producing CNTs as one of the products. It is measured to have a yield rate higher than 75% and produces CNTs with fewer structural defects [69]. In laser ablation, another commonly used approach first discovered in 1996, a transition metal doped graphite rod is vaporized in an oven at 1200 °C. Although this method only results in a 70% yield rate, which is slightly lower than that of the arc discharge method, it can produce SWCNTs with higher purity [107].

Apart from the plasma-based synthesis mentioned above, chemical vapor deposition (CVD) proved to be reliable and capable of achieving large-area, highly ordered and isolated CNTs in 1996. The substrate is made of mesoporous silica containing embedded iron nanoparticles and heated to 700 °C. Acetylene mixed in nitrogen is pumped into the chamber and decomposes to carbon atoms. The deposition of carbon atoms on the prepared substrate forms CNTs [108].

### 2.3. Graphene

Graphene is an atomically thick sheet of carbon atoms, discovered through the exfoliating of graphite in 2004 [72,109]. On account of the great electronic, mechanical, thermal and optical properties, e.g., high mechanical strength, electronic mobility, and optical transparency, graphene is well-suited for electrodes, transistors, batteries, and supercapacitors [110,111,112,113].

Similar to carbon dots, graphene can also be synthesized using various top-down and bottom-up strategies. On the one hand, mechanical exfoliation [84], liquid-phase exfoliation [83], and the unzipping of CNTs [114] are typical examples of top-down methods, sharing the working principle in common that larger precursors are destroyed for nano-sized graphene. Therefore, products fabricated from top-down approaches depend on precursors to a large extent [115]. Mechanical exfoliation is the earliest proposed method, where graphene was obtained from graphite [116,117]. The exfoliation can be divided into transverse and longitudinal according to the direction of force applied on the material surface [118]. Another exfoliation method is liquid-phase exfoliation, which was discovered in 2008 by Hernandez [119]. It consists of multiple steps, i.e., dispersing graphite in organic solutions, exfoliating the graphene, and purifying the product [120]. The schematics of liquid-phase exfoliation and mechanical exfoliation are shown in Figure 1g,h, respectively. From another point of view, considering the fact that CNTs are rolled graphite sheets, producing graphene by unzipping CNTs is easy to understand [121], as shown in Figure 1i. The unzipping step refers to cutting the internal bonds in the structure of CNTs and can be achieved by processes such as chemical attack, plasma etching, and laser irradiation [122,123]. On the other hand, the bottom-up synthesizing approaches include CVD, laser-assisted methods, and epitaxial growth [124]. CVD suggests that graphene films grow on metal substrates at high temperature, while graphene can also have epitaxial growth on SiC wafers, Cu/Ni, and Pt films. The high cost and complexity of operation are the two major challenges for bottom-up methods [125,126,127].

### 2.4. Stretchability

For the purpose of making use of carbon-based materials in soft robotic devices with collectively high yield and tensile strength, integrating carbon nanomaterials or composites with soft polymeric substrates is the most general and convenient scheme [128]. Various fabrication techniques have been utilized to achieve the incorporation, e.g., CVD [129], solution-based [130], and printing methods [131], which will be discussed respectively in this subsection.

It has been presented that graphene can be directly grown on soft substrates through CVD. Firstly, the PI substrate is prepared by coating liquid PI on glass and baking it several times at different temperatures. Secondly, Cu ink containing Cu nanoparticles is coated on the PI substrate. Last, plasma-enhanced CVD (PECVD) is used for graphene growth on Cu ink [132].

**Figure 2:**
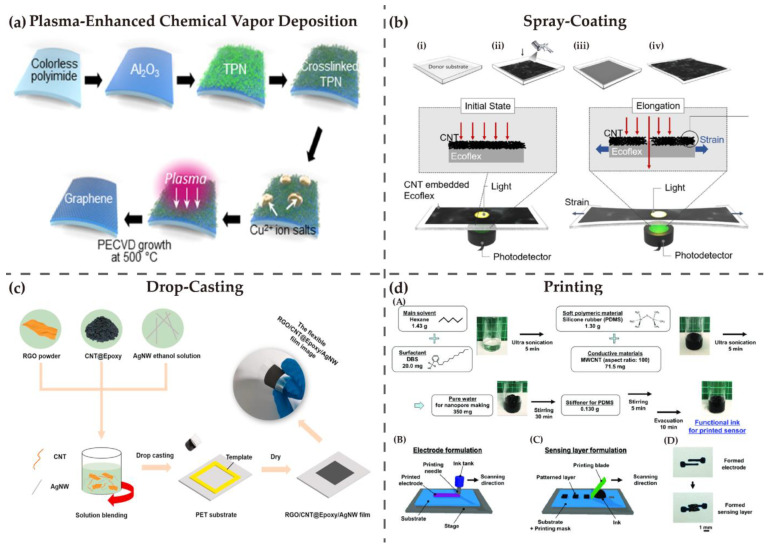
Fabrication techniques for merging carbon–based materials with soft substrate: (**a**) Use PECVD for direct graphene growth on flexible substrates. Reproduced from [136] with permission, copyright 2019, ACS. (**b**) Spray–coating for MWCNT–embedded Ecoflex films: (i) Using petri dish as the substrate; (ii) Spray–coating of CNT–IPA solution on petri dish; (iii) Pouring of Ecoflex on the substrate; (iv) Schematic of the MWCNT–embedded Ecoflex film. Reproduced from [133] with permission, copyright 2019, ACS. (**c**) Drop–casting for preparing flexible rGO/CNT/AgNW films. Reproduced from [135] with permission, copyright 2021, Elsevier. (**d**) Use printing technologies for fabricating pressure sensors: (**A**) Preparation process of MWCNT–PDMS ink; (**B**) Electrode formulation by printing; (**C**) Sensing formulation by printing; (**D**) Images of printed electrode and sensing layer. Reproduced from [137] with permission, copyright 2020, John Wiley and Sons.

Drop-casting, spray-coating, and spin-coating are a series of solution-based methods. Gu et al. used spray-coating method to develop a CNT-embedded Ecoflex film and made the thin film into a wearable strain sensor. The fabrication process of the elastomer is shown in Figure 2b, and it is mainly composed of mixing CNTs and isopropyl alcohol (IPA), spray-coating of the CNT-IPA solution on a petri dish, and adding Ecoflex prepolymer to the CNT film. After curing the mixture for 3 h at room temperature, a CNT-embedded Ecoflex film is formed, provided with good stretchability and sensitivity for sensors [133]. Compared to spray-coating, drop-casting is easier to control and more suitable for fabricating small-area films [134]. Flexible graphene-based films with excellent electromagnetic interference were manufactured by Song et al. in 2021. As shown in Figure 2c, reduced graphene oxide (rGO), AgNWs and CNTs, acting as conductive fiber, were stirred in ethanol solutions, and then the solution mixture was drop-casted on PET substrate to form a flexible carbon-based film [135].

Furthermore, printing is considered to be a convenient deposition method. A flexible carbon-based film can be fabricated by inkjet-printing composite containing CNTs onto a PI substrate [138]. A wide range of printing technologies can be employed in different steps so that nearly the whole fabricating process can be accomplished through printing. The research group successively utilized inkjet printing, insulator printing, and aerosol jet printing to produce the substrate, make the electrodes, and print SWCNT ink onto the PI substrate so as to form a fully printed low-voltage SWCNT CMOS inverter [139]. As shown in Figure 2d, printing by functional ink containing MWCNTs and PDMS can be a method to fabricate flexible sensor [137].

Other than incorporating carbon materials with soft substrates, the assembly method can also contribute to the better stretchability and performance of devices. Hu et al. enhanced the stretchability simply by prestretching the VHB tape, a kind of polyacrylate polymer. The film electrodes were prepared by spray-coating CNTs onto stretchable films. After assembling films with electrodes onto the prestretched tape and releasing the tape, the electrodes will be wrinkled, therefore equipping the device with higher stretchability [140].

## 3. Sensing with Carbon Materials

**Figure 3:**
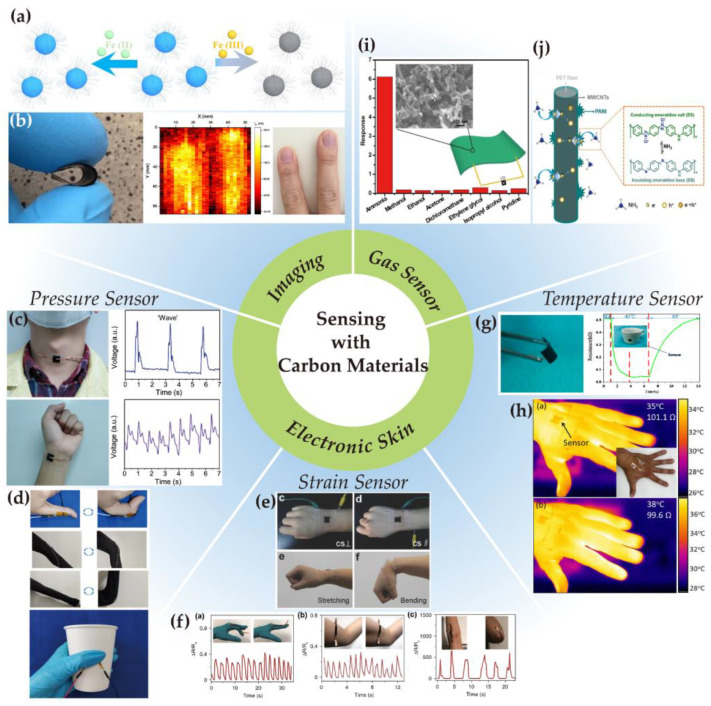
Applications of carbon–based sensors: (**a**) N–doped carbon dots for Fe(III) sensing and imaging in living cells. Reproduced from [153] with permission, copyright 2021, Elsevier. (**b**) Flexible polymer–CNT thermal sensor. Reproduced from [154] with permission, copyright 2018, ACS. (**c**) Highly sensitive and flexible pressure sensor built with carbon black. Reproduced from [155] with permission, copyright 2019, ACS. (**d**) Flexible capacitive pressure sensor with carbon fiber electrodes. Reproduced from [156] with permission, copyright 2021, ACS. (**e**) Flexible strain sensor based on carbonized conductive crepe paper. Reproduced from [32] with permission, copyright 2018, John Wiley & Sons. (**f**) Flexible carbon–based strain sensor with faster response. Reproduced from [157] with permission, copyright 2021, Springer Nature. (**g**) Flexible temperature sensor made of reduced graphene oxide. Reproduced from [158] with permission, copyright 2018, MDPI. (**h**) Flexible CNT–based temperature sensor with bio–compatibility. Reproduced from [159] with permission, copyright 2020, Elsevier. (**i**) Highly sensitive and flexible NH_3_ sensor. Reproduced from [160] with permission, copyright 2016, Elsevier. (**j**) Flexible MWCNT–based NH_3_ sensor, showing the sensing mechanism. Reproduced from [161] with permission, copyright 2021, Elsevier.

Sensors usually serve as significant detection tools that equip soft robots with biomimicking sensations and perceptions by converting different types of signals, e.g., physical and chemical signals, into electrical ones. They are responsible for reacting to stimuli as well as digitizing, quantifying, and visualizing the changes of targeting variables. As wearable electronics and smart devices have been hot topics in recent years, flexible and stretchable sensors are in great demand for research [141]. Functional materials widely used in flexible sensors include TMDCs [142], MXenes [143], NWs [144], and colloidal QDs [145]. Meanwhile, tremendous progress has been made in carbon-based flexible sensors [146], involving sensing toward pressure, strain, temperature, and humidity [147,148]. The rapid developing pace reveals the carbon material’s promising capabilities in easy processing, excellent electrical conductivity, and high sensing sensitivity [149]. For example, the addition of carbon nitride nanosheets in a hydrogel-based flexible sensor contributes to better tensile strength and toughness, providing hydrogel with better integration of its features [150]. Moreover, owing to the low toxicity and biocompatibility of carbon materials, carbon-based sensors especially excel in biological applications [151,152]. A few typical examples of flexible sensors based on carbon materials will be given in this section, and the categories to be discussed are listed in Figure 3.

As flexible and stretchable sensors always need to be bent, stretched, or compressed for at least hundreds of thousands of cycles, the mechanical robustness is a significant factor for a soft sensor. It can be enhanced by improving current synthetic approaches. Meanwhile, when sensing depends on the resistance, capacitance, and other electrical properties, power units are necessary and will decrease the convenience and portability of sensors. In this way, self-powered sensors can have great competence. More efforts are needed so that it can have more beneficial applications in electronic skin and outdoor research.

### 3.1. Imaging

Bioimaging is a form of biosensing and refers to visualizing biological activity, which helps with detecting and framing internal structures of cells, tissues and organisms [162]. Due to the excellent electroluminescent and photoluminescent effect, carbon dots are proposed to possess great chemical, biological and optical properties, and thus exhibit excellent performance and potential in nanomedicine [163]. The observed fluorescence emission wavelength of carbon dots falls in a wide range of 375–550 nm [164,165]. The features of light emission, attachment to cell surface, and bio-friendliness render carbon dots capable of imaging and biolabeling of bacteria and cells, and at the same time not influencing the health of the observer [166,167,168]. In a recent research study in which carbon dots were employed as agents for in vivo imaging, it was observed that the agents were excreted from the mouse several hours after injection, confirming the biosecurity of carbon dots [169]. As Figure 3a shows, prepared N-doped carbon dots can selectively detect Fe(III) in a water solution [153]. Furthermore, functionalizing carbon dots to make selective response to specific ions, e.g., Cr(VI), Fe(III) and Cu(II), has been evaluated to enhance the sensitivity and selectivity respectively by more than 12 and 7 times [170]. Based on their imaging and sensing performance, carbon dots can be built into flexible fluorescence sensing platforms, fluorescent nanoprobes, and can also be extended to cancer cell imaging, cancer marker detection, and early warning of tumor [171,172,173,174,175]. Moreover, it has been proved that the aggregation of carbon dots in solution will mostly cause the quench of fluorescence. Since the fluorescence capability is especially significant in imaging, the quenching effect of the carbon dots concentration ought to be prohibited, which can be achieved by avoiding π-π stacking between molecules from controlling the molecular conformation [176].

In addition, a flexible imaging system can also be realized through the integration of CNTs. As shown in Figure 3b, poly(vinyl alcohol) and CNTs were combined to fabricate flexible thermal detectors, which makes use of the photo-thermoelectric effect of the composite and paves the way toward thermal imaging and other biomedical applications [154]. Similar to the condition of carbon dots, when applying CNTs to in vitro and in vivo imaging, aggregated CNTs appear to obtain lower fluorescence intensity and lower toxicity than dispersed CNTs [177]. In this way, finding out the moderate degree of aggregation can provide proper fluorescence and relatively low toxicity, which is important in biologically relevant sensing and imaging.

### 3.2. Electronic Skin

Electronic or epidermal skin is an important aspect of wearable electronics with a focus on the real-time monitoring of human physiological signals, enabling tactile sensations for soft robots [178]. Due to the fact that the sensors embedded in electronic skin monitor various types of signals and gather a huge amount of information, it brings much convenience to daily health monitoring and medical diagnosis. As electronic skin has to conform to the movement trends of human epidermis, the used materials need to withstand twisting and stretching while providing a similar Young’s modulus to human skins and tissues. Varieties of sensors with different functions, including pressure sensors, strain sensors, and temperature sensors, are integrated to bring capability to simulate the “feeling” of actual skin [179]. Simultaneously, good biocompatibility and a certain degree of durability are necessary for electronic skin since it is a device in contact with human skin and will be exposed to long-term and repetitive movements [180].

Considering that detecting human health needs several vital physiological indicators, the electronic skin should be provided with multiple sensing capabilities. Firstly, it can be accomplished by applying a sensor array containing different types of sensors in one flexible substrate, which is a common fabrication strategy [181]. Alternative methods to integrate various sensing in one single sensor have also been found. In the multifunctional sensor, the CNT/PDMS film served as a sensing layer, and the sensor could react to external pressure and temperature change [182].

#### 3.2.1. Pressure Sensor

The basic sensing mechanism of pressure sensors is converting the change of physical pressure into electrical signals, and the transduction can be carried out in different forms, e.g., piezoresistivity, piezoelectricity, triboelectricity and capacitance [183]. Taking the piezoresistive pressure sensor as an example, it suggests that the applied pressure will change the size of the contact area, thus causing the change of the resistance value [184].

According to a study in 2019, a pressure sensor, with a wide working range of 0–30 kPa and high sensitivity of 51.23 kPa^−1^, can be easily fabricated by evenly dropping carbon black onto airlaid papers for several times. The sensor presented to be elastic and flexible due to the porous network structure of the airlaid paper, and the number of stacked layers influenced the sensing sensitivity and flexibility. Attaching this sensor to a human neck or wrist can achieve voice recognition or pulse monitoring, as shown in Figure 3c [155]. Different from the multilayer flat structure, the microsphere structure may provide better sensing performance. The pressure sensor consisting of two copper electrodes and sandwiched CNT-wrapped PDMS microspheres was discovered to have fast response and good durability through dynamic loading tests. Compared to the plane-to-plane structure, its plane-to-microsphere structure presents a more significant decline in electrical resistance change in response to applied pressure. As the working principle of such sensing is achieved by the observation of material resistance change in response to pressure change, more dramatic resistance decline of the plane-to-microsphere structure results in higher sensitivity, about 15 times higher than the one of the plane-to-plane structure [185].

Capacitive pressure sensors are also widely used, relying on the capacitance change caused by external pressure stimuli. A carbon-based printed capacitive sensor was fabricated in 2021, where carbon fiber was 3D printed to be electrodes, presenting short response time of 60 ms, and excellent stability of withstanding 1000 cycles. Figure 3d shows its multiple applications with human motions, including bending of the thumb, knee, and elbow, as well as different hand-holding patterns [156].

#### 3.2.2. Strain Sensor

Considering that both strain and pressure sensors sense the changes caused by external strain or pressure applied on the surface, strain sensors analogously depend on varying trends of resistivity, capacitance, and piezoelectricity. Among these categories, resistive and capacitive types are more frequently used [186].

Huge efforts have been made in devising various fabrication methods in order to make enhancements to the sensing performance, mainly in terms of flexibility, sensitivity, durability, and working range. By depositing Ag nanoparticles and CNTs on PDMS, a strain sensor can be created with good stability, high sensitivity, and stretchability of 98.5% [187]. Three-dimensional printing also provides an alternative approach with low cost, easy operation and high efficiency [188]. Printing MWCNT-polymer composites layer by layer and curing them though ultraviolet can produce a pyramid configuration. A strain sensor based on this printed structure was found to enjoy a high sensitivity of 8.939, a wide detectable strain range of 0.01% to 60% and a high durability of 10,000 cycles [147].

By 2018, a strain sensor fabricated on carbonized conductive crepe paper was measured to reach high flexibility, high durability, and fast response time at 115 ms [32]. Photographs of the sensor attaching to the wrist for sensing its bending and stretching can be seen in Figure 3e. Subsequently in 2021, the response time was further reduced to 60 ms. The strain sensor was made up of carbon black embedded flexible film, as shown in Figure 3f, along with a wide workable stretching range of up to 160% [157].

#### 3.2.3. Temperature Sensor

In human health monitoring, one of the most considerable indicators is body temperature, so temperature sensors are considered essential components in electronic skin. Temperature sensors are categorized as four types with regard to their sensing units, namely thermistors, transistors, capacitive, and thermocouple devices. Sensing through thermistors is widely used in wearable devices due to its high sensitivity, and its sensing function depends on whether the resistance changes positively or negatively with the temperature [189]. For example, printing CNT-graphene oxide ink onto PET substrate can form an ultrathin and ultrasensitive temperature sensor, where CNTs in proper proportion provide the sensor with a negative temperature coefficient [190].

Progress has been made in improving the sensing accuracy and independency from the application of massive external strain in sensing temperature changes. The measured inaccuracy reduced to 1 °C with strain up to 60% in 2018 by applying an innovative voltage readout scheme, and the stretchable circuits were made from SWCNTs [191]. Soon after, in 2021, a newly designed aligned electrospun carbon nanofiber film was utilized in temperature sensor. The flexible sensor presents high responsive to temperature among multiple stimulus, high accuracy near to thermometers, fast response time, and outstanding durability [192].

It is worth mentioning that the change in thermal resistance can also be utilized as an indicator of the binding of biomolecules, which gives rise to novel wearable biomedical and healthcare devices. In particular, sensors using screen-printed graphite electrodes have enabled the accurate and low-cost diagnosis of cardiovascular diseases based on the binding of cardiac biomarker troponin and molecularly imprinted nanoparticles [193].

### 3.3. Gas Sensor

Gas sensing is of the resistive type, as the specific gas changes the surface conductance when interacting with the sensing layer [194]. Carbon-based gas sensors have a wide range of testable objects, such as NO_2_, H_2_S, CO_2_, and NH_3_ [195]. Taking the NO_2_ detector as an instance, the resistance of the sensor showed a difference between the conditions with air and NO_2_, making the sensor acquire selectivity of the tested gas, high response of 24.82%, and long-term sensing stability. At the same time, the sensor was fabricated by depositing a polypyrrole and nitrogen-doped MWCNT composite onto the PI substrates so that it achieved excellent stretchability [196]. Furthermore, a NO_2_ gas sensor reported in 2022 reached better sensing and stretching performance. The gas sensor was made from depositing MWCNTs with CeO_2_ powders onto silicon rubber. Its response time to NO_2_ demonstrated to decrease from 36.6 s to 25.6 s with stretching the sensor from 0% to 100%, and the detecting range is quite large [197].

Moreover, printing technology renders possibilities to fully print gas sensor with low cost and low power consumption. A thin film transistor containing CNTs was printed though an aerosol jet printer, and the side gates were printed by Ag ink. The sensor was provided with high sensitivity to H_2_S, high response at room temperature, and extremely low power consumption, demonstrating great competitive strength [198]. Similar fabrication strategies can be applied to sensors for other gases, such as NH_3_. The gas sensor fabricated by aerosol jet printing SWCNT ink appeared to have extremely high response and outstanding selectivity of NH_3_ [199].

## 4. Soft Robotic Actuation with Carbon Materials

Actuators are devices that respond to various stimuli, including heat [200], pressure [201], light [202], humidity [203], electricity [204], and magnetism [205]. Carbon-based soft robotic actuators mainly consist of carbon nanomaterials and flexible substrates. For example, a multi-responsive actuator made up of a graphene oxide film and a layer containing mixed CNTs and PDMS was proposed in 2018, and it could produce reversible deformation under thermal, light, and humidity conditions [206]. Another actuator had displacement in response to voltage stimulation or light irradiation, in which CNTs were blade-coated to achieve orderly direction. It demonstrated bending deformation larger than 10 mm under external stimulus, having great inspiration for bionic soft robotics [207].

Thanks to their flexibility and stretchability, soft robotic actuators behave better than their rigid peers in fields of biomimicry and soft robotics [208,209], as do the actuators containing carbon nanomaterials. This section presents actuation reacting to heat, light irradiation, and piezoelectricity.

**Figure 4:**
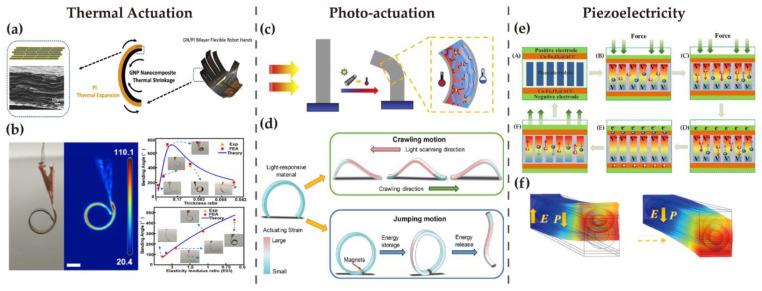
Typical actuation mechanisms for carbon–based soft robots: (**left**) (**a**) Flexible robotic hand based on graphene nanoplates and PI, demonstrating the bending mechanism of electro–thermal actuators. Reproduced from [210] with permission, copyright 2019, Elsevier. (**b**) Graphene–based electro–thermal actuator. Reproduced from [211] with permission, copyright 2022, ACS. (**middle**) (**c**) CNT–based light–driven fiber actuator. Reproduced from [212] with permission, copyright 2021, Elsevier. (**d**) Worm–like photo–actuated soft robot that can realize crawling, squeezing, and jumping. Reproduced from [213] with permission, copyright 2019, John Wiley & Sons. (**right**) (**e**) Flexible self–charging supercapacitor based on carbon cloth, showing the self–charging process: (**A**) Schematic of the supercapacitor composition; (**B**) Reaction to external force and generation of piezoelectric field; (**C**) Migration of electrolyte ions and charging; (**D**) Continuous migration of electrolyte ions and charging after external force is ceased; (**E**) New equilibrium after migration; (F) Piezoelectric field disappears and the supercapacitor returns to initial state. Reproduced from [214] with permission, copyright 2020, Elsevier. (**f**) Piezo–actuator containing aligned CNTs. Reproduced from [215] with permission, copyright 2021, Elsevier.

Although these reported soft robotic actuators present an outstanding and obvious response to external stimuli, most of them are still on the centimeter or millimeter length scale, as shown in Figure 4, which remains to be greatly improved in the future. The internal structural design, use of materials, and the actuating mechanisms are among the factors that could be taken into account. Further, the minimization of soft actuators can benefit their applications in bio-related areas, especially under in vivo circumstances.

### 4.1. Thermal Actuation

Considering that the actuating mechanism of thermal actuation is converting thermal energy into kinetic energy and creating motion, materials involved should demonstrate expansion or contraction in response to thermal stimulation. For instance, hydrogel is regarded as a promising candidate for wearable electronics due to its reaction to thermal change [216]. In thermal actuation, the stimulation is usually provoked by change in ambient temperature or heat generated by electricity [217].

According to the introduced actuating mechanism, when applying voltage to conductive films, thermal-induced expansion or contraction will take place as the current flow heats up the films. The degree of deformation is closely related to the coefficient of thermal expansion (CTE), which is a material property. In a multi-layer structure, the mismatch of CTEs between the layers will result in bending, as shown in Figure 4a. The carbon-based film is often treated as the conductive layer because of its excellent electrical conductivity, as well as being responsible for transferring heat between multiple layers due to its extreme sensitivity to heat. With relatively lower CTE, the carbon layer is less stretched than polymeric layers, leading to the bending deformation of carbon-based flexible films [210,218]. For instance, a typical electro-thermal actuator can be made up of three layers, namely the CNT layer, Kapton^®^ layer, and shape memory polymer (SMP) layer. The applied voltage heats the CNT layer and heat transfers, making the SMP layer become flexible and finally inducing the bending [219]. Electro-thermal actuation excels in terms of the low applied voltage since a composite layer of CNTs and PDMS can achieve a large deformation at as low as 8 V bias [220]. In addition, the bending radius is influenced and controlled by multiple factors, involving the applied voltage, the layer thickness, and the CTE of different materials. In this case, the actuator can accomplish specific robotic motions, such as grasping and releasing objects [210].

### 4.2. Photo-Actuation

Photo-actuation is an easily implemented actuation method and basically relies on either the photochemical reactions or the photothermal effects of materials [221,222]. Although photothermal actuators similarly make use of the thermal sensitivity of materials with electro-thermal ones, they behave better at enabling contactless manipulation [223].

The fabrication of photo-actuators can be carried out by merging two films with different CTEs together, such as SWCNTs and polycarbonate. The photo energy absorbed by SWCNT film converts to thermal energy, resulting in the deformation of different extents [224]. Similarly, composites of liquid crystal elastomer and CNTs can be constructed into a light-powered soft robot. When the composite film was exposed to light irradiation, the surface facing the light had higher temperature than the opposite side, prompting the film to contract unevenly and therefore bend toward the light source. On the basis of the bending motion, the tiny robot can perform various locomotion under different illumination modes like a worm, including crawling, contraction, and jumping, as is shown in Figure 4d [213]. The carrying capacity has also been exceedingly enhanced in 2022, being reported to uphold loads more than 4600 times of the actuator’s own weight. The composite film created by depositing CNTs onto a liquid crystal elastomer fiber displayed excellent photo-actuating behavior, and therefore it can be employed as artificial muscles [212].

It has also been proposed that precise functions other than functional movements, such as printing, can be realized by properly designing and utilizing photo-actuators. For example, a type of photo-actuated pen array made from CNT-PDMS composite realized massively parallel molecular dip-pen nanolithography. The pen would locally expand and print ink on the substrate when specific ones were exposed to light [225].

### 4.3. Piezoelectricity

Piezoelectric materials, by nature, are able to convert mechanical strain into electricity energy. This capability of energy transduction can be utilized in piezo-resistive strain sensors, nanogenerators, supercapacitors, and applications revolving around energy harvesting. Interestingly, inverse piezoelectric materials geometrically respond to voltages applied, which can be used to build piezo-actuators for soft robots [226,227].

Piezo-actuators are established on voltage-induced motion or the deformation of inverse piezoelectric materials. Advances have been made to improve their actuating performance. Merging the MWCNT solution into polyvinylidene fluoride and distributing in the axial direction contribute to better bending capability, measured as bending to 24 μm in response to an electric field of 4 V/μm [215]. Types and quantities of materials can make a difference; for instance, adding a different amount of few-layer graphene into silicone rubber with different tensile modulus will change the conductivity and thus influence the actuating motion [228].

Due to the energy conversion allowed by the piezoelectric effect, piezoelectric batteries can also realize self-charging simply through bending or patting themselves. In nanogenerators, carbon-based materials are significant components of electrodes. Taking the one published in 2019 as an example, both the cathode material and anode material were carbon treated for higher electronic conductivity [229]. In addition to nanogenerators, carbon materials can also be used as flexible electrodes in supercapacitors, one of the energy harvesting applications. Graphene oxide-based electrodes were proved to have high rate capability in a supercapacitor proposed in 2020 [230]. Another flexible self-chargeable supercapacitor proposed in 2021 adopted electrodes fabricated by directly growing Co-Fe_2_O_3_ particles on activated carbon cloth. The assembled supercapacitor can charge itself with great durability of bending for 420 cycles, and the self-charging process is displayed in Figure 4e [214].

## 5. Conclusions and Outlook

In conclusion, we summarized the foundational development, fabrication methods of carbon-based materials, and their vital roles in novel soft robotic sensors and actuators in this review. Different from their rigid counterparts, these flexible and stretchable devices have made possible a great number of soft robotic applications. However, more in-depth study is yet to be done in order to tackle the following challenges:

(1) The further miniaturization of devices.

This requires a complete and compact integration of perception and motion systems of soft robots by joint efforts from the communities of materials science, chemistry, physics, mechanical engineering and electrical engineering. In particular, the physical dimensions of flexible and stretchable sensing unit will have to shrink to a greater extent such that the sensors become fully implantable and biocompatible for in vivo perception. As for the motion or actuation component, a more rapid response to external stimuli is needed for robotic manipulation in order to perform intricate operations and surgeries.

(2) The mechanical robustness of devices.

While carbon materials, such as CNT yarns, possess outstanding tensile strength, the collective mechanical robustness of the entire device needs to be further improved from a synthetic perspective. More sophisticated flexible carbon composite materials remain to be discovered to exhibit high mechanical yield stress, as well as outstanding electronic properties, such as carrier mobility for carbon-based semiconductors in applications for state-of-the-art optoelectronic devices and field effect transistors.

(3) The power efficiency of sensing and actuation unit.

Importantly, we can only make self-sustained devices when they are fully self-powered and untethered. It is worth mentioning that the power supply system plays a vital role here in that the device performance and portability is greatly hindered by very limited battery lifetime and power density. This could potentially lead to completely untethered ultralow power electronic skins with built-in flexible power supply.

Once these issues are properly resolved, the compliant carbon materials could then turn into a state-of-the-art brain–computer interface (BCI), micro-/nano-wearable and implantable sensors, and smart textiles. We expect further evolution and extraordinary technical improvement in the use of carbon-based materials in state-of-the-art entirely stretchable electronics devices and soft robots, in terms of design, fabrication and applications, in the next 10 to 20 years.

## Figures and Tables

**Table 1:** Fabrication methods of carbon dots, CNTs and graphene.

Fabrication Methods	Products	Advantages	Disadvantages	Ref.
Laser ablation	Carbon dots, CNTs	Rapid process and effectiveness; Facile control of production yield by varying laser wavelength	Sophisticated equipment setup; High temperature and pressure; Poor size control; Not suitable for mass production	[75,76]
Arc discharge	Carbon dots, CNTs	Highly fluorescent carbon dots; High water solubility; High-quality CNT production	Sophisticated equipment setup; High temperature and pressure; Difficult product purification	[77,78]
Microwave-assisted method	Carbon dots	Tunable particle hydrophobicity; Fast process; Low cost; Environmental protection	Poor size control; Low production yield	[79]
Template method	Carbon dots	Better size control of carbon dots; size was more uniform; High water solubility; Facile tuning of emission color by adjusting temperature and oxidation time	High cost; Low production yield; Complicated product separation	[80,81]
Chemical Vapor Deposition	CNTs	Even growth on irregular surfaces; High purity; High production yield; Suitable for mass production	Sophisticated equipment setup; High temperature requirement;	[82]
Liquid-phase exfoliation	Graphene	Moderate quality and cost; Suitable for mass production	Low production yield; Small graphene lateral dimensions	[83]
Mechanical exfoliation	Graphene	Scalable and sustainable; Low structural defects; Low cost; High production yield	Fragmentation effects; Relatively low efficiency	[84,85]
Unzipping of CNTs	Graphene	Facile synthetic processes; Low cost; Well-defined nanoribbon geometry; Excellent candidates for electronics	Complex synthetic mechanism; Low-throughput characterization	[86,87]

## Data Availability

Not applicable.
